# A new mathematical model of multi-faced COVID-19 formulated by fractional derivative chains

**DOI:** 10.1186/s13662-022-03677-w

**Published:** 2022-01-21

**Authors:** Ibtisam Aldawish, Rabha W. Ibrahim

**Affiliations:** 1 Department of Mathematics and Statistics, College of Science, IMSIU (Imam Mohammad Ibn Saud Islamic University), Riyadh, Saudi Arabia; 2 IEEE: 94086547, Kuala Lumpur, 59200 Malaysia

**Keywords:** Fractional calculus, Fractional differential equation, Fractional derivative chains, COVID-19, Transformations

## Abstract

It has been reported that there are seven different types of coronaviruses realized by individuals, containing those responsible for the SARS, MERS, and COVID-19 epidemics. Nowadays, numerous designs of COVID-19 are investigated using different operators of fractional calculus. Most of these mathematical models describe only one type of COVID-19 (infected and asymptomatic). In this study, we aim to present an altered growth of two or more types of COVID-19. Our technique is based on the ABC-fractional derivative operator. We investigate a system of coupled differential equations, which contains the dynamics of the diffusion between infected and asymptomatic people. The consequence is accordingly connected with a macroscopic rule for the individuals. In this analysis, we utilize the concept of a fractional chain. This type of chain is a fractional differential–difference equation combining continuous and discrete variables. The existence of solutions is recognized by formulating a matrix theory. The solution of the approximated system is shown to have a minimax point at the origin.

## Introduction

It is very significant to present the mathematical simulations of infectious viruses for a better assessment of their survival, constancy, and control. As the traditional methodologies of mathematical representations do not conclude the high gradation of truthfulness to describe these diseases, fractional calculus, including fractional differential, integral, and hybrid (mixed integral-differential and differential-integral) equations, was introduced to avoid such difficulties (for some recent works, see [1–4]). All fractional operators have various applications in practical areas like construction problems, optimization issues, artificial intelligence, optics, medical identification, automation, biology, and numerous other fields. In the previous few decades, fractional calculus has been utilized in the mathematical description of biological phenomena. This is for the reason that arbitrary calculus can clarify and establish the existence of custom properties of numerous materials truthfully compared to ordinary simulations. For further presentations about fractional calculus in biostatistics, bioinformatics, biomedical and biomathematical systems, we refer to the recent papers [5–9].

Henceforward, the above-mentioned information is presented and studied from numerous viewpoints, namely we present a qualitative study, optimization theory, and numerical analysis. Therefore, investigators extended the traditional calculus to the generalized calculus modeling, using different mathematical procedures. Nowadays, many researchers have deliberated mathematical representations of COVID-19 under the fractional calculus (see the very recent publications in this direction [10–17]). Using the current data from European and African countries, Atangana and Araz offered different statistical analyses [18–20]. Moreover, Atangana and Araz [21] presented a numerical mathematical modeling system utilizing the Newton polynomial. Other approaches can be found in [21–30].

We investigate the growth of two or more coexisting types of COVID-19. The ABC-fractional derivative operator formalizes our procedure. We deal with a system of coupled differential equations, which contains the dynamics of the diffusion between infected and asymptomatic populations. The outcome is accordingly associated with a macroscopic rule for the individuals. Moreover, this analysis is formulated with the concept of a fractional chain. This type of chain is a fractional differential–difference equation combining continuous and discrete variables. The existence of solutions is established by formulating a matrix theory. Some numerical results are illustrated in the sequel.

The rest of the paper is organized as follows: Sect. 2 presents the methodology that will be used in our study; Sect. 3 describes the results and discussion of the suggested model; Sect. 4 provides the conclusion and directions for future works.

## Methodology

### ABC-definition

The elementary viewpoint and appearances of fractional calculus and its applications are realized in numerous assessments and evaluations. Most studies on the fractional calculus contain kernels. For example, the main difference between the Caputo differential operator, the Caputo–Fabrizio operator [31], and others is that the Caputo differential operator is associated with a power law, the Caputo–Fabrizio differential operator is modified by employing an exponential growth term. Atangana–Baleanu differential operator is formulated by suggesting the generalized Mittag-Leffler function [32].

#### Definition 2.1

Let $\Delta ^{\alpha }$, $\alpha \in (0,1)$ be the Atangana–Baleanu differential operator of order *α* of a function *g* having the structure

$$ \Delta ^{\alpha } g(t)= \frac{D(\alpha )}{1-\alpha } \int _{0}^{t} g'( \tau ) \Sigma _{\alpha } \biggl(\frac{-\alpha }{1-\alpha } (t-\tau )^{\alpha } \biggr) \,d\tau , \quad t\in [0,\infty ), $$

where $D(\alpha )$ denotes a normalization function, while $E_{\alpha }$ indicates the Mittag-Leffler function

$$ E_{{\alpha }}(\eta )=\sum_{{n=0}}^{\infty }{ \frac{\eta ^{n}}{\Gamma (\alpha n+1 )}}.$$

Associated with $\Delta ^{\alpha }$, the ABC integral is realized by

$$ \Lambda ^{\alpha }g(t)= \frac{(1-\alpha ) }{D(\alpha )}g(t)+ \frac{\alpha }{D(\alpha )\Gamma (\alpha )} \int _{0}^{t} g(\tau ) (t- \tau )^{\alpha -1} \,d\tau . $$

#### Example 2.2

The function $g(t)=t^{\kappa }$ has the ABC integral

$$ \Lambda ^{\alpha } t^{\kappa }= \frac{(1-\alpha ) }{D(\alpha )} t^{\kappa }+ \frac{\alpha \Gamma (\kappa +1)}{D(\alpha )\Gamma (\kappa +1+ \alpha )} t^{\kappa +\alpha }. $$

In our study, since we focus on the approximated solutions, we assume that $D(\alpha ) \rightarrow 1$, for all $\alpha \in (0,1)$.

### Infected dynamics

We assume that $\mathbb{T} (t) $ is the total number of infected individuals, which characterizes the sum of two numbers, the customary infected individuals $\chi (t)$ and those involved in the asymptomatic transmission $\Upsilon (t)$, so that $\mathbb{T} (t) =\chi (t) + \Upsilon (t)$. We take into account that $\chi (t)$ includes people who were previously sick. Consequently, there are frequency functions, combining *χ* and ϒ. In this study, we assume that $\mathbb{T}$ contains two sets of variables: continuous time variables and multiple discrete variables, namely numbers of infected and asymptomatic. Since COVID-19 has multiple faces, we may assume that $\mathbb{T}$ has chain descriptions in both categories of the variables. Two faces of COVID-19 have the description $\mathbb{T}(m,n,t,s)$, where $(m,n) \in \mathbb{N}^{2}$ are the discrete variables and $(s,t) \in \mathbb{R}^{2}$, $s\leq t$ are the continuous variables. One can extend the functional $\mathbb{T}$ into three faces as $\mathbb{T}(m,n,k, t,s,\ell )$, and so on for finite faces, when we have $\mathbb{T}(m_{1},\dots ,m_{j}, t_{1},\dots ,t_{j})$, where $(m_{1},\dots ,m_{j}) \in \mathbb{N}^{j}$ are the discrete variables and $(t_{1},\dots ,t_{j}) \in \mathbb{R}^{j}$.

### ABC-fractional chain

In general, a chain is an integrable differential–difference equation joining at least one continuous variable and one discrete variable. The first derivative of this chain is used to suggest a system of differential equations. A fractional chain was formulated for the first time by using the Riemann–Liouville differential operator (see [33]). Based on this idea, we improve the fractional chain using a fractional differential operator for several continuous and discrete variables, namely the ABC-fractional differential operator.

In this part, we use the above information to define the ABC-fractional chain. We deal with a two-dimensional functional $\mathbb{T}$. That is, $\mathbb{T}$ has two discrete variables, as well as two continuous variables. Similarly, for the extension to higher dimension. Define the ABC-fractional chain as follows:

2.1 $$ \Delta _{t}^{\alpha } \mathbb{T}(m,n,t,s)= \biggl( \frac{1}{\mathbb{T}(m+1,n,t,s)-\mathbb{T}(m-1,n,t,s)} \biggr), $$

where $\mathbb{T}(m,n,t,s)$ is a function depending on discrete and continuous variables $(m,n)\in \mathbb{N}^{2}$ (discrete variables) and $(t,s )\in \mathbb{R}^{2}$ (continuous variables), and $\Delta _{t}^{\alpha }$ is Atangana–Baleanu differential operator of order *α* with respect to the continuous variable *t*. Moreover, we consider the lowest order of (2.1) to be structured by

2.2 $$\begin{aligned} &\Delta _{s}^{\alpha } \mathbb{T}(m,n,t,s) \\ &\quad= \frac{ \mathbb{T}(m+2,n,t,s)}{( \mathbb{T}(m+1,n,t,s)- \mathbb{T}(m-1,n,t,s))^{2}( \mathbb{T}(m+2,n,t,s)- \mathbb{T}(m,n,t,s))( \mathbb{T}(m,n,t,s)- \mathbb{T}(m-2,n,t,s))} \\ &\qquad{}- \frac{ \mathbb{T}(m-2,n,t,s)}{( \mathbb{T}(m+1,n,t,s)- \mathbb{T}(m-1,n,t,s))^{2}( \mathbb{T}(m+2,n,t,s)- \mathbb{T}(m,n,t,s))( \mathbb{T}(m,n,t,s)- \mathbb{T}(m-2,n,t,s))} \end{aligned}$$

where $\Delta _{t}\Delta _{s}\mathbb{T}=\Delta _{s}\Delta _{t} \mathbb{T}$. To present the dynamic system, we have the following construction: by using (2.1), we have

2.3 $$\begin{aligned} \begin{aligned} &\mathbb{T}(m-2,n,t,s)= \biggl( \mathbb{T}(m,n,t,s) - \frac{1}{\Delta _{t}^{\alpha }\mathbb{T}(m-1,n,t,s)} \biggr), \\ &\mathbb{T}(m-1,n,t,s)= \biggl(\mathbb{T}(m+1,n,t,s) - \frac{1}{\Delta _{t}^{\alpha }v_{m,n}} \biggr), \\ & \mathbb{T}(m+2,n,t,s)= \biggl(\mathbb{T}(m,n,t,s) + \frac{1}{\Delta _{t}^{\alpha }\mathbb{T}(m+1,n,t,s)} \biggr). \end{aligned} \end{aligned}$$

Substituting (2.3) into (2.2), we get the nonlinear system

2.4 $$ \begin{aligned} &\Delta _{s }^{\alpha } \Phi =\Delta _{t }^{\alpha } \bigl(\Delta _{t }^{\alpha } \Phi \bigr) +2 \bigl( \Delta _{t }^{ \alpha }\Phi \bigr) ^{2}\Delta _{t }^{\alpha }\Psi , \\ &\Delta _{s }^{\alpha }\Psi = - \Delta _{t }^{\alpha } \bigl(\Delta _{t }^{\alpha }\Psi \bigr)+2 \bigl( \Delta _{t }^{\alpha }\Psi \bigr) ^{2} \Delta _{t}^{\alpha }\Phi , \end{aligned}$$

where $\Phi :=\mathbb{T}(m,n,t,s)$ and $\Psi :=\mathbb{T}(m+1,n,t,s)$. In view of (2.1), we have the transmission information

2.5 $$\begin{aligned} & \bigl(\mathbb{T}(m,n,t,s), \mathbb{T}(m+1,n,t,s) \bigr)\rightarrow \bigl( \mathbb{T}(m+1,n,t,s), \mathbb{T}(m+2,n,t,s) \bigr), \end{aligned}$$

2.6 $$\begin{aligned} & \bigl(\mathbb{T}(m-1,n,t,s),\mathbb{T}(m,n,t,s) \bigr)\rightarrow \bigl( \mathbb{T}(m,n,t,s), \mathbb{T}(m+1,n,t,s) \bigr). \end{aligned}$$

Hence, we get the transformations

2.7 $$ \begin{aligned} \begin{pmatrix} \Phi \\ \Psi \end{pmatrix}\rightarrow \begin{pmatrix} \Psi \\ \Phi +\frac{1}{\Delta _{t}^{\alpha } \Psi }\end{pmatrix} \end{aligned}$$

and

2.8 $$ \begin{pmatrix} \Phi \\ \Psi \end{pmatrix} \rightarrow \begin{pmatrix} \Psi -\frac{1}{\Delta _{t}^{\alpha }\Phi } \\ \Phi \end{pmatrix} . $$

System (2.4) represents the dynamics of multi-face of COVID-19, where *m* is the number of sick people on the recent face, while *n* is for the previous face. We suppose that the previous face is eliminated or terminated completely. But, there are some countries, still suffering from the two faces, where the previous face has not completely disappeared, yet. In this case, we suggest another dynamical system.

### Shifted dynamic system

Clearly, Eqs. (2.1) and (2.2) impose the discrete equation of the structure

2.9 $$ \bigl(\mathbb{T}(m,n,t,s)-\mathbb{T}(m+1,n+1,t,s) \bigr) \bigl( \mathbb{T}(m+1,n,t,s)- \mathbb{T}(m,n+1,t,s) \bigr)+\flat =\wp , $$

where ♭ and ℘ are fixed constants. Equation (2.9) indicates the KdV-type and pKdV-type equations. Also, (2.1) and (2.2) imply the symmetry of (2.9). Hence, the conclusion is that there exists a function $\Xi (t ,s )$ such that

2.10 $$ \Delta _{t }^{\alpha }\Xi =0,\qquad \Delta _{s }^{\alpha }\Xi =0, $$

where

2.11 $$\begin{aligned} \Xi (t,s) :=& \bigl(\mathbb{T}(m,n,t,s)-\mathbb{T}(m+1,n+1,t,s) \bigr) \bigl( \mathbb{T}(m+1,n,t,s)- \mathbb{T}(m,n+1,t,s) \bigr) \\ &{}+\flat -\wp . \end{aligned}$$

Consequently, we have the shifted quantities

2.12 $$ \phi =\mathbb{T}(m,n+1,t,s),\qquad \psi =\mathbb{T}(m+1,n+1,t,s). $$

Thus, we obtain the shifted dynamical system

2.13 $$ \begin{aligned} &\Delta _{s}^{\alpha } \phi = \Delta _{t}^{\alpha } \bigl( \Delta _{t}^{\alpha } \phi \bigr) +2 \bigl(\Delta _{t}^{\alpha } \phi \bigr)^{2} \Delta _{t}^{\alpha } \psi , \\ & \Delta _{s}^{\alpha } \psi = - \Delta _{t}^{\alpha } \bigl(\Delta _{t}^{ \alpha } \psi \bigr) +2 \bigl( \Delta _{t}^{\alpha } \psi \bigr)^{2} \Delta _{t}^{\alpha }\phi . \end{aligned}$$

Combining (2.4) and (2.13), we have

2.14 $$ (\Phi -\psi ) (\Psi -\phi )=\eth ,\qquad \eth :=\wp -\flat . $$

Equation (2.10) can be written in the up–down shifted form with respect to *m*, namely

2.15 $$\begin{aligned}& \bigl(\mathbb{T}(m+1,n,t,s) - \mathbb{T}(m+2,n+1,t,s) \bigr) \\& \quad {}\times \bigl(\mathbb{T}(m+2,n,t,s)-\mathbb{T}(m+1,n+1, t,s) \bigr)=\eth \end{aligned}$$

and

2.16 $$ \bigl(\mathbb{T}(m-1,n,t,s)-\mathbb{T}(m,n+1,t,s) \bigr) \times \bigl( \mathbb{T}(m,n,t,s)-\mathbb{T}(m-1,n+1,t,s) \bigr)=\eth . $$

Utilizing Eq. (2.1), we get

2.17 $$ \mathbb{T}(m+2,n,t,s)=\Phi + \frac{1}{\Delta _{t }^{\alpha }\Psi },\qquad \mathbb{T}(m+2,n+1, t,s)=\phi +\frac{1}{\Delta _{t }^{\alpha }\psi }, $$

and

2.18 $$ \mathbb{T}(m-1,n,t,s)=\Psi +\frac{1}{\Delta _{t }^{\alpha }\Phi }, \qquad \mathbb{T}(m-1,n+1,t,s)= \psi +\frac{1}{\Delta _{t }^{\alpha }\phi }. $$

From (2.15) and (2.16), we have

2.19 $$ \biggl(\Psi -\phi - \frac{1}{\Delta ^{\alpha }_{t} \psi } \biggr) \biggl( \Phi -\psi - \frac{1}{\Delta ^{\alpha }_{t} \Psi } \biggr) =\eth $$

and

2.20 $$ \biggl(\Psi -\phi - \frac{1}{\Delta ^{\alpha }_{t} v} \biggr) \biggl( \Phi -\psi - \frac{1}{\Delta ^{\alpha }_{t} \phi } \biggr) =\eth . $$

System (2.13) indicates the dynamics of multi-face COVID-19, where *m* is the number sick people on the recent face and *n* is the number of the previous face, which is not terminated yet. Both systems (2.4) and (2.13) can be generalized into *j* faces. Moreover, one can generalize the above systems by using the 1D-parametric structure as follows:

2.21 $$\begin{aligned}& \bigl( \mathbb{T}(m+\nu ,n,t,s)-\mathbb{T}(m+1+\nu ,n, t,s) \bigr) \\& \quad {}\times \bigl( \mathbb{T}(m+\nu +1,n,t,s)-\mathbb{T}(m+ \nu ,n,t,s) \bigr) =\eth _{\nu }, \end{aligned}$$

where *ν* is an arbitrary integer. Similarly, for the shifted system. From (2.21), we have the system

2.22 $$ (\Phi -\psi ) (\Psi -\phi )=\eth _{\nu }, $$

where $\Phi =\mathbb{T}(m+\nu ,n,t,s), \Psi =\mathbb{T}(m+\nu +1,n, t,s)$ and $\mathbb{T}(m+\nu ,n+1,t,s)=\phi , \mathbb{T}(m+\nu +1,n+1, t,s)=\psi $. In addition, 2D-parametric structure can be realized by considering a new parameter for *n* to become

2.23 $$ \begin{aligned}[b] & \bigl( \mathbb{T}(m+\nu ,n+\mu ,t,s)-\mathbb{T}(m+1+ \nu ,n+\mu +1t,s) \bigr) \\ &\quad {}\times \bigl( \mathbb{T}(m+\nu +1,n+\mu ,t,)s-\mathbb{T}(m+ \nu ,n+\mu +1,t,s) \bigr) =\eth _{\nu ,\mu }, \end{aligned} $$

which implies the system

2.24 $$ (\Phi -\psi ) (\Psi -\phi )=\eth _{\nu ,\mu }. $$

## Results and discussion

In this section, we investigate the stability of systems (2.4) and (2.13). We have the following results for system (2.4), which can be extended to system (2.13).

### Theorem 3.1

*Consider system* (2.4). *Then system* (2.4) *has a minimax point*.

### Proof

System (2.4) can be reduced to the matrix system

$$\begin{aligned}& \begin{pmatrix} \Delta _{s }^{\alpha }\Phi \\ \Delta _{s }^{\alpha }\Psi \end{pmatrix} = \begin{pmatrix} a & 2b \\ 2c & -a \end{pmatrix} \begin{pmatrix} \Delta _{t }^{\alpha }\Phi \\ \Delta _{t}^{\alpha }\Psi \end{pmatrix} \\& \bigl(a:= \Delta ^{\alpha }, b:= \bigl(\Delta _{t }^{\alpha } \Phi \bigr)^{2}, c:= \bigl(\Delta _{t }^{\alpha } \Psi \bigr)^{2} \bigr). \end{aligned}$$

The above system can be approximated at the fixed point of $\Delta _{t }^{\alpha }\Phi $ and $\Delta _{t}^{\alpha }\Psi $ to obtain the linear system

3.1 $$\begin{aligned} \begin{pmatrix} \Delta _{s }^{\alpha }\Phi \\ \Delta _{s }^{\alpha }\Psi \end{pmatrix}= \begin{pmatrix} a & 2b \\ 2c & -a \end{pmatrix} \begin{pmatrix} \Phi \\ \Psi \end{pmatrix}. \end{aligned}$$

The eigenvalues of this system are

3.2 $$\begin{aligned} \lambda _{1,2}= \pm \sqrt{a^{2}+4bc}, \qquad a^{2} + 4 b c>0, \end{aligned}$$

which correspond to the eigenvectors

$$ V_{1,2}= \biggl( -\frac{(-a \pm \sqrt{a^{2} + 4 b c})}{2 c}, 1 \biggr).$$

Hence, the critical point is a saddle point (minimax point) satisfying

3.3 $$\begin{aligned} \max (\Phi ,\Psi _{0})= (\Phi _{0},\Psi _{0})=\min (\Phi _{0},\Psi ). \end{aligned}$$

 □

### Corollary 3.2

*The solution of system* (2.13) *satisfies*

3.4 $$\begin{aligned} (\min \sup )_{m,n,t,s} (\Phi ,\Psi ) = ( \sup \min )_{m,n,t,s} ( \Phi ,\Psi ). \end{aligned}$$

### Proof

By Theorem 3.1, the origin is a solution of system (2.13) satisfying

3.5 $$\begin{aligned} \max (\Phi ,\Psi _{0})= (\Phi _{0},\Psi _{0})=\min (\Phi _{0},\Psi ), \end{aligned}$$

where $(\sup )_{m,n,t,s} (\Phi ,\cdot )$ is the lower value in $\operatorname{dom}(\Psi )$ and $( \min )_{m,n,t,s} (\cdot ,\Psi )$ is the upper value in $\operatorname{dom}(\Phi )$. Hence, we obtain the desired assertion. □

### Remark 3.3

**Figure 1:**
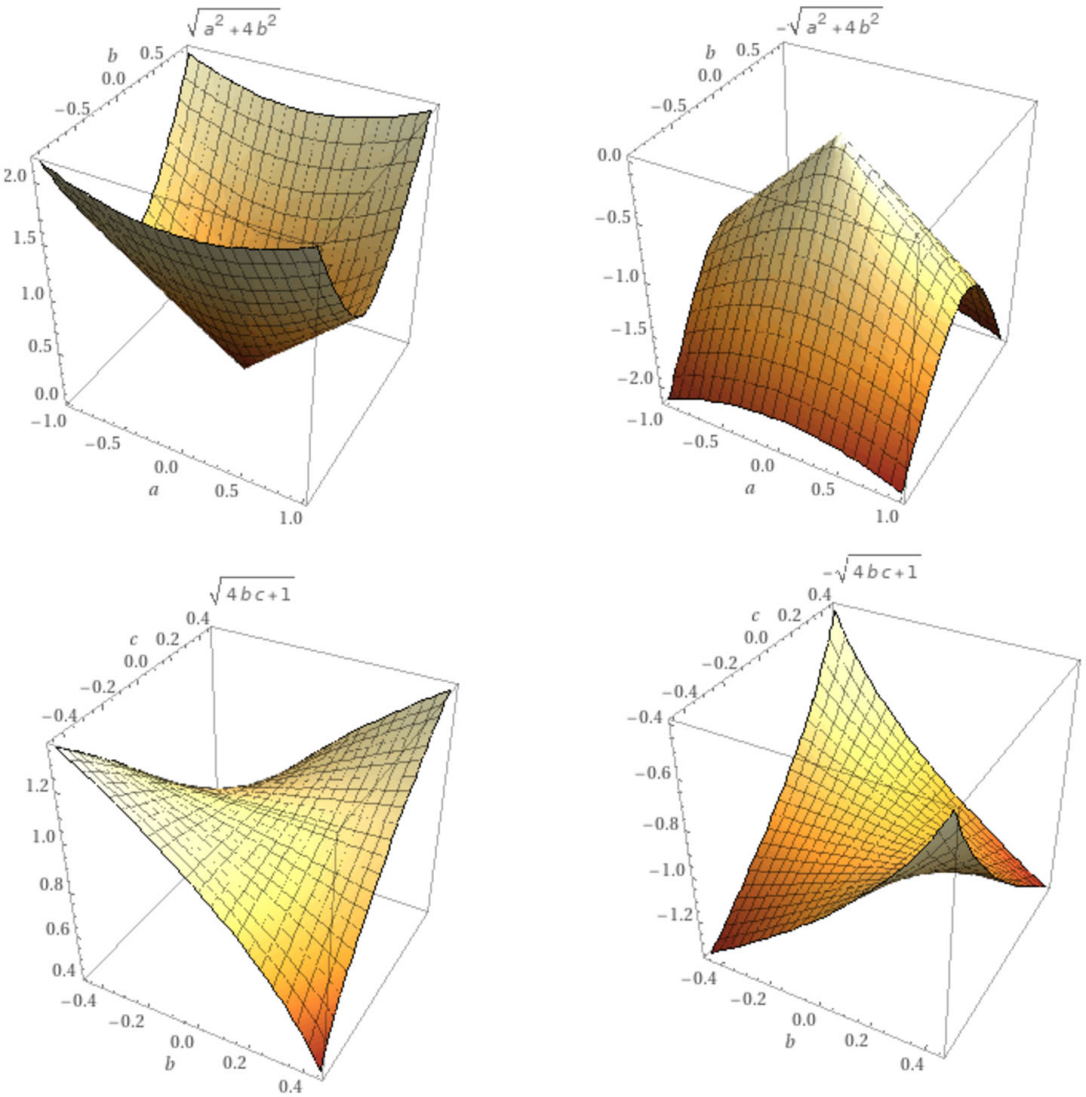
Parametric plot of the eigenvalues of system (2.13). The first row indicates the global minimum, when $b=c$, while the second row represents the local minimum, when $a=1, b\neq c$

- Note that this point represents the transmission from one face to another of the coronavirus. The ordinary case of system (3.1) is known as the Wilson–Cowan system, which is utilized in formulating neuronal or cell population [34].
- The rate of expansion can be evaluated by the formula [34]
  3.6 $$\begin{aligned} R:= \frac{\partial t}{\top } *\complement , \end{aligned}$$
  where ∁ is the is the speed of waves from the origin, $\partial t=t-s$, and ⊤ indicates the period of the periodic solution (the number of the recent face of the coronavirus). Wilson and Cowan evaluated the average of the speed by letting $\complement =22.4\text{ mm}/\text{s}$. Using system (3.1), the rate can be recognized by a fractional derivative
  3.7 $$\begin{aligned} R_{\alpha }(\Phi ):= \frac{\Delta _{s }^{\alpha }\Phi }{\top } *22.4, \quad R_{\alpha }(\Psi ):= \frac{\Delta _{s }^{\alpha }\Psi }{\top } *22.4. \end{aligned}$$
- In view of Theorem 3.1, system (3.7) has a minimum point. Figure 1 shows two important cases, a global minimum and a local minimum.

### Example 3.4

Consider system (2.4), and set

$$ \upsilon (s):= \bigl(\Delta _{t }^{\alpha } \bigl(\Delta _{t }^{ \alpha }\Phi \bigr) +2 \bigl( \Delta _{t }^{\alpha }\Phi \bigr) ^{2} \Delta _{t }^{\alpha }\Psi \bigr)$$

and

$$ \omega (s):= \bigl(- \Delta _{t }^{\alpha } \bigl(\Delta _{t }^{\alpha } \Psi \bigr)+2 \bigl( \Delta _{t }^{\alpha }\Psi \bigr) ^{2}\Delta _{t}^{ \alpha }\Phi \bigr).$$

Then the solution can be formulated by the integral system of equations, with the initial condition $\Phi _{0}=0,\Psi _{0}=0$,

3.8 $$\begin{aligned} &\Phi = \frac{(1-\alpha ) }{D(\alpha )}\upsilon (s)+ \frac{\alpha }{D(\alpha )\Gamma (\alpha )} \int _{0}^{s} \upsilon ( \tau ) (s-\tau )^{\alpha -1} \,d\tau , \end{aligned}$$

3.9 $$\begin{aligned} &\Psi = \frac{(1-\alpha ) }{D(\alpha )}\omega (s)+ \frac{\alpha }{D(\alpha )\Gamma (\alpha )} \int _{0}^{s} \omega ( \tau ) (s-\tau )^{\alpha -1} \,d\tau . \end{aligned}$$

**Figure 2:**
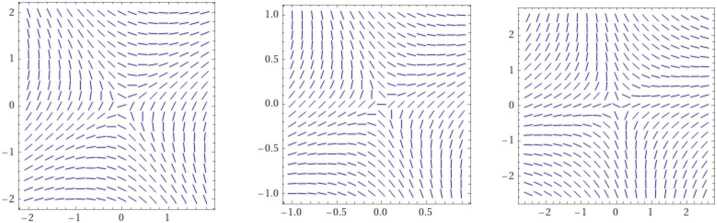
Slope field of solutions of the system (2.4). The solution is approximated at the maximum case, when $\alpha \rightarrow 1$, where $\alpha \in (0,1]$, *x*-axis is Φ and *y*-axis is Ψ

Figure 2 presents the behavior of the solution for different values of $\alpha \in (0,1]$. The behavior of the solution shows the minimax point at the origin. The solution is approximated at the maximum case, when $\alpha \rightarrow 1$, by

3.10 $$\begin{aligned} &\Phi = \frac{1}{10 }c_{1} e^{-\sqrt{5} t} \bigl((5 + \sqrt{5}) e^{2 \sqrt{5} t} + 5 - \sqrt{5} \bigr) + \frac{c_{2} e^{-\sqrt{5} t} (e^{2 \sqrt{5} t} - 1)}{\sqrt{5}}, \end{aligned}$$

3.11 $$\begin{aligned} &\Psi = \frac{c_{1} e^{-\sqrt{5} t} (e^{2 \sqrt{5} t} - 1)}{\sqrt{5}}- \frac{1}{10 }c_{2} e^{-\sqrt{5} t} \bigl((\sqrt{5}-5) e^{2 \sqrt{5} t} - 5 - \sqrt{5} \bigr). \end{aligned}$$

## Conclusion

The minimax point theorem is one of the greatest significant consequences of the mathematical analysis theory. It indicates that there is a technique, which together minimizes the maximum loss (sick people) and maximizes the minimum improvement (healthy people). Roughly speaking, there is an approach, which normal people would take supposing the worst-case situation.

Summarizing the above analysis, we have formulated a new mathematical technique based on fractional calculus with the ABC-derivative operator. We formulated a system that satisfies multiple faces of the coronavirus. The total number is suggested as a continuous function of time, which is discrete in the number of faces. We used an approximation method to analyze the system. We recognized that the solution possesses a minimax point. This point indicates the termination of the recent face and realizes a new face of the corona virus.

## Data Availability

Data sharing not applicable to this article as no datasets were generated or analyzed during the current study.
